# Safety and Feasibility of Analgosedation for Electrochemotherapy of Liver Lesions

**DOI:** 10.3390/life13030631

**Published:** 2023-02-24

**Authors:** Roberto Iezzi, Alessandro Posa, Cosimo Tommaso Caputo, Davide De Leoni, Fabio Sbaraglia, Marco Rossi, Giampaolo Tortora, Luca Tagliaferri, Vincenzo Valentini, Cesare Colosimo

**Affiliations:** 1 Department of Diagnostic Imaging, Oncologic Radiotherapy and Hematology, Fondazione Policlinico Universitario A. Gemelli IRCCS, L.go A. Gemelli 8, 00168 Rome, Italy; 2 Department of Anesthesia and Intensive Care, Fondazione Policlinico Universitario A. Gemelli IRCCS, L.go A. Gemelli 8, 00168 Rome, Italy; 3 Department of Medical and Surgical Sciences, Fondazione Policlinico Universitario A. Gemelli IRCCS, L.go A. Gemelli 8, 00168 Rome, Italy

**Keywords:** electrochemotherapy, interventional oncology, ablation, liver cancer, sedation, analgesia

## Abstract

Interventional Oncology treatments grant low-risk mini-invasive alternatives to surgery for cancer patients. Percutaneous ablative therapies represent a cornerstone for treatment of liver cancer patients. Among these, a newly emerging one is represented by electrochemotherapy. Improvements in analgesia and sedation can nowadays offer optimal support for ablative procedures, serving as a valid alternative to general anesthesia. The intention of this retrospective monocentric study is to report our preliminary experience on feasibility and safety of electrochemotherapy for treatment of complex liver tumors unfit for thermal ablation, using analgosedation instead of general anesthesia. Five patients were enrolled in the study, undergoing electrochemotherapy under analgosedation. Mean procedural time and hospitalization time were recorded. Immediate post-procedural cone-beam CT showed complete coverage of the lesion without complications. One-month CT examination showed an overall response rate of 100% (four complete responses, one partial response). Electrochemotherapy under analgosedation seems to be a safe, feasible, and effective option for liver cancer patients not amenable to other ablative techniques.

## 1. Introduction

Interventional oncology (IO) offers image-guided procedures for cancer patients that can be considered as an alternative to medical and surgical procedures and also reduce peri-interventional risks thanks to their minimal invasiveness [1]. Since its advent, due to technical innovations and new devices, its role has expanded across a wide spectrum of disease sites, offering curative as well as palliative care for many types of cancer patients [2].

Percutaneous liver ablation has become a cornerstone of interventional oncology [3].

The last few decades have seen a rapid improvement in thermal ablation technology, with significant technical and procedural advancements that can enhance safety profiles and clinical outcomes [4].

In recent years, a new nonthermal tumor ablation modality is emerging, the so-called electrochemotherapy (ECT) [5]. A locally generated electrical field (electroporation) is used in this treatment in order to rapidly increase the permeability of tumor cells, making the cellular membranes more permeable to hydrophilic chemotherapy drugs (such as bleomycin) [6].

ECT effectiveness has been approved in head and neck tumors, subcutaneous or cutaneous lesions, cervix leiomyosarcoma, and breast cancer, whereas development is focused on treatment of deep-seated tumors, such as primary and secondary liver lesions [7,8,9,10,11,12].

However, when compared to other ablative options, ECT is hampered by the requirement for general anesthesia and muscular relaxation, both of which may increase the risks of the procedure, particularly in geriatric patients [13]. Additionally, many individuals cannot undergo general anesthesia, particularly if they suffer from serious cardiovascular or respiratory comorbidities.

In the last years, analgosedation developed as an efficacious and well-tolerated approach in several fields, representing a goal-oriented procedure, offering optimal and safe support for IO procedures, and a valid alternative to general anesthesia [14].

Based on this background, the aim of our study was to report our preliminary experience on performing electrochemotherapy for treatment of liver tumors using analgosedation, instead of general anesthesia, focusing on feasibility and safety.

## 2. Materials and Methods

The Institutional Review Board granted ethical approval for the study in accordance with the 1990 Declaration of Helsinki and its later amendments. All patients provided their written informed consent.

This is a retrospective observational single-center pilot study on the employment of electrochemotherapy for the treatment of complex liver lesions—not suitable for thermal ablation—under analgosedation.

Patients were selected for locoregional treatments after thorough discussion during a multidisciplinary tumor board meeting. Within 30 days before treatment, all patients were submitted to physical examination and blood tests, as well as radiological examinations. Pulmonary function assessment was also performed due to potential bleomycin-related pulmonary toxicity. Diagnosis of hepatocellular carcinoma (HCC) was made on the basis of LiRADS scores or biopsies, whereas all metastatic patients were enrolled after histological diagnosis [15].

All consecutive patients with primary and/or metastatic liver lesions treated with electrochemotherapy under analgosedation from May to September 2022 were included in our pilot study. Indications for electrochemotherapy usually include unresectable primary or secondary hepatic lesions, with liver-only or liver-dominant metastatic disease, with less than 25% tumor load, and good performance status (ECOG status 0–1; Eastern Cooperative Oncology Group), not amenable for thermal ablation, mainly due to complex location. Contraindications for thermal ablation were: (1) Peri-tumoral vicinity of the common, left, or right hepatic bile duct, (2) peri-hepatic critical structures that could not be distanced using interventional dissection methods, (3) the abutment or encasement of a single remaining major portal or systemic vein following surgery, (4) gallbladder vicinity, and (5) intra-abdominal free-surface/subcapsular or subdiaphragmatic lesion location.

The following data were collected: Patient characteristics (age, sex, comorbidities), tumor characteristics (histology, largest diameter, location), and procedure-related information (number of probe-needles, procedural time, technical success, ablation necrosis extent, complication rates, as well as hospitalization length).

Procedural time was defined as the time interval between the first procedural image acquisition and the last control scan. Technical success was defined as the correct placement of probe needles around the tumor and the execution of the electric impulse protocol after bleomycin infusion. Early response was evaluated at a 1-month CT follow-up.

### 2.1. Procedural Steps

All procedures were performed on an inpatient basis under analgosedation and strict surgical asepsis. For antibiotic prophylaxis, 2 g of cefazolin was administered intravenously before the procedure. Procedures were performed by an experienced interventional radiologist with 20 years of experience in performing ablative procedures.

Freehand electrodes were used for the percutaneous therapy, placing a maximum of 6 probes using the CliniporatorTM devices (IGEA Ltd., Modena, Italy) based on size, configuration, and localization of the target lesion. Therapy planning was software-based (XperGuide, Philips), using a parallel positioning of the probes at a distance from each other of no more than 2.5 cm.

Treatment planning was performed based on preprocedural CT and/or MR images. Intraprocedural needle electrode placement was performed under US and CBCT guidance. The Cliniporator TM device was used to produce electrical pulses. This device is able to generate square wave pulses with an amplitude variating from 1 to 5000 Hz. Soon after electroporation, the intravenous administration of bleomycin was performed (15 mg/m^2^ body surface area) in a time frame of less than 5 min. According to the standardized criteria, after bleomycin administration, electric pulses were generated from 8 to 40 min in order to achieve optimal tissue drug delivery [16]. Since the impulses are delivered between pairs of electrodes, the device calculated the voltage to be applied for each pair of electrodes independently. Combining the proper electric field for each pair ensured that the tumor was completely covered.

### 2.2. Analgosedation Protocol

All patients were treated under deep sedation during spontaneous breathing, with a total intravenous analgesic and sedative drug administration. Standard monitoring, based on an electrocardiogram (EKG), non-invasive blood pressure (NiBP), and oxygen saturation (SaO2), was obtained.

Before starting the procedure, the pacoretients were premedicated with intravenous midazolam 0.01–0.02 mg/kg. In the operating theater, an infusion of propofol (TCI Schnider model) and remifentanil (TCI Minto Model) was started. Propofol infusion was usually titrated to reach a BISpectral Index between 60 and 70, while remifentanil was maintained at 1–2 ng/mL during the first step of the procedure and 2–3 ng/mL during the painful phase of the treatment. In order to guarantee a quick detection of potential apnea/hypopnea episodes, close control of capnography was carried out. Once the procedure was concluded, anesthetics drugs were discontinued: their rapid offset was compatible with a rapid discharge by the Recovery Room. Post-procedural analgesia was obtained with acetaminophen and, if required, ketoprofen [17,18,19].

### 2.3. Post-Procedural Follow-Up

Hospitalization length was calculated in days. Perioperative morbidity and mortality included major/minor complications and death, respectively, occurring during the first 7 days. Complications were defined using the classification of complications from CIRSE and based on Common Terminology Criteria for Adverse Events (CTCAE) version 5.0 [20,21]. Complications were considered minor if classified as CTCAE grade 1 or 2, and major if classified as CTCAE grade 3 or 4. Moreover, complications were also classified based on their onset as immediate (occurring in the first 24 h after the procedure), periprocedural (occurring in the first 30 days after the procedure) or delayed (occurring after 30 days from the procedure) [22].

For evaluation of complications after the procedure, all patients underwent hemoglobin, serum aminotransferase, liver function tests within 24 h, 7 days, and 30 days after the procedure, and US within 48–72 h after the procedure. Clinical evaluation, as well as laboratory tests and multiphasic spiral CT studies (64-row CT Lightspeed VCT scanner, GE Medical Systems; unenhanced, arterial, portal, and late phases; slice thickness 0.6 mm; contrast flow rate 4 mL/s) were performed one month after the procedure to evaluate responses to therapy, according to the Modified Response Evaluation Criteria in Solid Tumors (mRECIST) criteria, and to detect/exclude complications. Lesion-based treatment success was assessed using the mRECIST criteria in terms of complete remission (CR), partial remission (PR), stable disease (SD), and progressive disease (PD).

In the case of death, it was determined whether the death was causally related to the target lesion or not.

### 2.4. Statistical Analysis

Statistical analysis was performed by using SAS version 9.4 (SAS, Cary, NC, USA). Continuous variables were reported as mean ± standard deviation. Categorical variables were reported as absolute numbers and percentages.

## 3. Results

**Figure 1:**
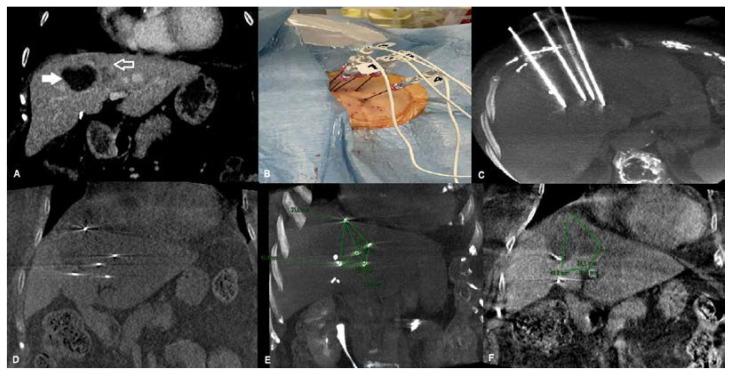
An 81-year-old female affected by recurrence of liver metastasis from pancreatic cancer after percutaneous ablation and SBRT, treated with percutaneous ECT. (**A**) Pre-treatment coronal CT reconstruction showing dishomogeneous enhancing tissue consistent with metastatic liver recurrence (contoured arrow) at the periphery of the hypodense post-ablation and post-SBRT area (white arrow). (**B**) Percutaneous placement of the five electrodes. (**C**) Unenhanced cone-beam CT MPR and MIP reconstruction showing the five electrodes positioned in the target lesion. (**D**) Unenhanced cone-beam CT coronal reconstruction showing the tip of the five electrodes in place. (**E**) Unenhanced cone-beam CT coronal reconstruction showing the tip of the five electrodes in place and the post-processing measurements underlining the geometrical relationship between each electrode. (**F**) Contrast-enhanced post-ECT cone-beam CT coronal reconstruction showing the ablation area. Legend: ECT: Electrochemotherapy; SBRT: Stereotactic body radiation therapy; CT: Computed tomography; MPR: Multiplanar reconstruction; MIP: Maximum intensity projection.

From May to September 2022, 42 percutaneous liver ablations were performed in our Institution. Among these, five were ECT treatments (mean age: 67.2 ± 11.3 years; range: 56–81; 3 males) and were considered suitable for our retrospective study. Of the five liver lesions treated with ECT, two (40%) were primary cancers (HCC), whereas the last three were secondary cancers. Four of them were naive lesions, whereas the last one was a post-ablative and stereotactic body radiation therapy (SBRT) metastatic liver recurrence of pancreatic cancer (Figure 1).

The mean largest diameter of the lesions was 37.6 mm (range: 26–54 mm; standard deviation (DS): 11 mm). In four patients, 17-Gauge (G) 16-cm-long electrodes with a 4-cm active portion were used, whereas in the last patient, 21-G 20-cm-long electrodes with a 3-cm active portion (IGEA, Carpi, Italy) were used. The direction of access and electrode position were determined by the operator.

Reasons for thermal ablation contraindication were as follows: Two lesions were located adjacent to the common hepatic bile duct, two lesions were located near peri-hepatic critical structures (bowel) that could not be distanced using interventional dissection methods, and the last lesion was located adjacent to the gallbladder and on the intra-abdominal free-surface of the liver.

In the first three patients, general anesthesia was contraindicated due to heart diseases, whereas in the last two patients, analgosedation was preferred to general anesthesia due to patients’ comorbidities and old age.

The procedure was feasible and technically successful in all patients. Enhanced cone-beam computed tomography (CBCT) performed at the end of the procedure revealed complete coverage of the lesions with no evidence of bleeding or other peri-interventional complications. The mean procedural time was 75.6 min (range: 61–91 min–DS: 11.6 min).

Soon after the intervention, the patients were awake, conscious, and oriented, and none of them claimed having felt any pain or discomfort during the procedure. In addition, no patient complained about muscle soreness during the following days. No hematologic or pulmonary toxicity was observed. No erythema or edema occurred at the site of electrode access. Patients were discharged between 24 and 48 h post-intervention, with a mean hospitalization length of 2.4 days (DS: 0.54 days).

No major or minor complications occurred. All blood parameters returned to normal range within two weeks. No patient complained of complications or adverse events during the post-interventional home care.

At one-month CT exams, a mean necrotic diameter of 43.6 mm (range: 35–52 mm; DS: 6.6 mm) was obtained, with an overall response rate of 100%, a complete response in four lesions and one partial response with persistence of vital tissue <30%.

## 4. Discussion

In multimodal treatment of liver neoplasms, locoregional minimally invasive image-guided procedures are becoming more and more significant. In detail, local ablative therapies (LAT) expanded the therapeutic spectrum in multimodal liver cancer therapy, opening up new options in terms of therapy tolerability and compliance [23].

Percutaneous locoregional therapies can be divided into thermal (radiofrequency (RFA) or microwave ablation (MWA) and cryoablation) and nonthermal options. Literature confirmed that tumor size (greater than 3 cm) and the presence of large (3 mm or more) abutting vessels significantly affect the efficacy of RF ablation due to heat loss secondary to the perfusion-mediated tissue cooling within the area [24]. These limitations were overcome by MWA. However, hyperthermia-based technologies (RFA and MWA) are also limited by their applicability due to tumor location. In particular, ablative procedures on sub-glissonian lesions or lesions located next to the gallbladder or the portal vein, or adjacent to the gastrointestinal tract are considered at major risk of periprocedural complications [25]. Up to 30% of small-sized neoplastic lesions may not be suitable for ablative procedures due to their high-risk location [12,26].

As an alternative to thermal ablation, electrochemotherapy (ECT) was introduced for improving ablative results, being characterized by nonthermal mechanism, that means without heat sink effect and harm to vessels, with more apoptosis than necrosis, based on the pulsed electrical field instead of joule heating, with consequent easier to simulate the physics and better plannable intervention [27].

A locally produced electrical field (electroporation) is used in this process to temporarily increase the permeability of tumor cells, making the cellular membranes more permeable to hydrophilic chemotherapy drugs (bleomycin). The increased permeability to chemotherapy helps obtain a reduction of systemic drug dosages, with a boosted local effect. Moreover, electroporation leads to vasoconstriction, which causes hypoxic cell injury and extended retention of the cytostatic drugs in the neoplastic tissue.

The development of a new pulse generator and long needle electrodes allowed the application of ECT for the treatment of liver lesions. A few recent papers demonstrated the feasibility, safety, and efficacy of the percutaneous approach to electrochemotherapy for the treatment of HCC [11,12,28,29].

However, ECT is still limited by the longer preparation time due to the several electrodes that are placed around the tumor in parallel orientation to each other to ensure a successful ablation. To better execute this kind of procedure, advancements have been focused on probe placement assistance by developing image-guided navigational software and devices [30]. In our experience, the use of navigational software allowed us to perform a safe and accurate procedure in about 1 h, which is the reported time for standard thermal ablation (RFA or MWA).

The applicability of ECT in the treatment of liver lesions is also limited by the need of general anesthesia and muscle relaxation because of the electrical impulses, as well as EKG synchronization to ensure impulse delivery in the refractory phase of the heart, as reported in the literature [31,32]. However, general anesthesia is associated with higher costs, a longer hospital stay, and the anesthesia team might not be available when needed. Furthermore, general anesthesia is also not suitable in many patients, in particular, due to severe cardiovascular or respiratory comorbidity or old age. In the last years, the use of analgosedation in interventional oncology has exponentially increased, allowing performing complex locoregional treatments also in patients suffering from serious illnesses and comorbidities [14]. However, to perform analgosedation, it is necessary to have knowledge and great awareness of the complications these drugs can lead to [33].

In our clinical practice, we have the opportunity to work with dedicated anesthesiologists, well-trained in analgosedation for IO procedures. This facility allowed us to ask for a tailored analgosedation for ECT procedures in liver lesions as intraoperative management needs to take into account patients’ clinical conditions, angiography suite environment, and variability in pain exposure during the procedure. In our center, we usually provide deep sedation during spontaneous breathing, obtained with a total intravenous drug administration. This choice is due to improve global outcomes in a high-risk cohort of patients. The deep sedation indeed allows avoiding adverse events associated with intubation and mechanical ventilation. Furthermore, a total intravenous drug administration technique is suitable for simultaneous titration of both analgesia (remifentanil) and deep sedation (propofol).

The combination of drugs with intravenous administration usually allows having a predictable onset and duration of action, with collaborative patients during device insertion and adequate level of sedation and analgesia during the most delicate phase of electric pulse delivery. Analgosedation needs to be started before the procedure—in order to adequately prepare the patient—with repeated dosing to maintain a consistent, adequate level of sedation or analgesia during the procedure. An essential element of this approach is multi-parameter monitoring, above all, depth of sedation level and capnography.

In our experience, analgosedation allowed us to perform a safe, rapid, and effective procedure without serious intraoperative or postoperative adverse events. No relevant pain, muscle ache-like symptoms, or systemic side effects were observed.

Study limitations include its retrospective nature, the short observation period, and the small cohort of patients with a heterogeneous clinical history, neoplasm histology, and location, as well as inhomogeneity regarding previous treatments. Patients’ heterogeneity, however, shows the potential of ECT procedures for liver lesions, with a good variety of patients who could benefit from this treatment using analgosedation. Furthermore, to the best of our knowledge, this is the first paper reporting the use of analgosedation for liver ECT procedures, opening the door for future widening use and an indication of this treatment option also for liver lesions, overcoming previously perceived disadvantages/limitations avoiding the use of general anesthesia. It is well-known that these preliminary results warrant further validation with studies on larger populations, with longer follow-up, in order to also provide effective data on long-term local tumor control as well as long-term progression-free survival.

## 5. Conclusions

Our preliminary experience demonstrated that ECT performed under analgosedation represents a feasible, safe, effective, and valuable option for the treatment of primary or secondary liver lesions not amenable to other ablative techniques. Intravenous analgosedation was well tolerated in our patients, without significant pain or adverse events as well as complications, allowing for rapid post-interventional recovery.

## Data Availability

Not applicable.
